# The complete organellar genomes of the entheogenic plant *Psychotria viridis* (Rubiaceae), a main component of the ayahuasca brew

**DOI:** 10.7717/peerj.14114

**Published:** 2022-10-18

**Authors:** Alessandro M. Varani, Saura R. Silva, Simone Lopes, Jose Beethoven Figueiredo Barbosa, Danilo Oliveira, Maria Alice Corrêa, Ana Paula Moraes, Vitor F.O. Miranda, Francisco Prosdocimi

**Affiliations:** 1 Department of Agricultural and Environmental Biotechnology, School of Agricultural and Veterinarian Sciences, São Paulo State University (UNESP), Jaboticabal, São Paulo, Brazil; 2 Laboratory of Genetics and Molecular Biology, State University of Paraíba (UEPB), Campina Grande, Paraíba, Brazil; 3 Department of Plant Science (DFIT), Federal University of Roraima (UFRR), Boa Vista, Roraima, Brazil; 4 Laboratory of Bioprospection and Applied Ethnopharmacology, Faculty of Pharmacy, Federal University of Rio de Janeiro (UFRJ), Rio de Janeiro, Rio de Janeiro, Brazil; 5 Laboratório de Genômica e Biodiversidade, Instituto de Bioquímica Médica Leopoldo de Meis, Universidade Federal do Rio de Janeiro, Rio de Janeiro, Rio de Janeiro, Brazil; 6 Center for Natural Sciences and Humanities, Federal University of ABC (UFABC), São Bernardo do Campo, São Paulo, Brazil; 7 School of Agricultural and Veterinarian Sciences, Department of Biology, São Paulo State University (Unesp), Jaboticabal, São Paulo, Brazil

**Keywords:** Ayahuasca, Genome, Mitochondrion, Chloroplast, Comparative genomics, Phylogenomics, Entheogen, Psychotria viridis, Chacrona, Organelle

## Abstract

*Psychotria viridis* (Rubioideae: Rubiaceae), popularly known as *chacrona*, is commonly found as a shrub in the Amazon region and is well-known to produce psychoactive compounds, such as the N,N-dimethyltryptamine (DMT). Together with the liana *Banisteropsis caapi*, *P. viridis* is one of the main components of the Amerindian traditional, entheogenic beverage known as ayahuasca. In this work, we assembled and annotated the organellar genomes (ptDNA and mtDNA), presenting the first genomics resources for this species. The *P. viridis* ptDNA exhibits 154,106 bp, encoding all known ptDNA gene repertoire found in angiosperms. The *Psychotria* genus is a complex paraphyletic group, and according to phylogenomic analyses, *P. viridis* is nested in the Psychotrieae clade. Comparative ptDNA analyses indicate that most Rubiaceae plastomes present conserved ptDNA structures, often showing slight differences at the junction sites of the major four regions (LSC-IR-SSC). For the mitochondrion, assembly graph-based analysis supports a complex mtDNA organization, presenting at least two alternative and circular mitogenomes structures exhibiting two main repeats spanning 24 kb and 749 bp that may symmetrically isomerize the mitogenome into variable arrangements and isoforms. The circular mtDNA sequences (615,370 and 570,344 bp) encode almost all plant mitochondrial genes (except for the *ccmC*, *rps7*, *rps10*, *rps14*, *rps19*, *rpl2* and *rpl16* that appears as pseudogenes, and the absent genes *sdh3, rps2, rsp4, rsp8, rps11, rpl6*, and *rpl10*), showing slight variations related to exclusive regions, ptDNA integration, and relics of previous events of LTR-RT integration. The detection of two mitogenomes haplotypes is evidence of heteroplasmy as observed by the complex organization of the mitochondrial genome using graph-based analysis. Taken together, these results elicit the primary insights into the genome biology and evolutionary history of *Psychotria viridis* and may be used to aid strategies for conservation of this sacred, entheogenic species.

## Introduction

The Amazonian biodiversity is abundant though poorly studied and explored in the genomics age. One of the most sacred plants used as medicine by Amerindian tribes is the Amazonian *Psychotria viridis* Ruiz & Pav. (Rubioideae: Rubiaceae), a shrub that is popularly known as *chacrona* or *rainha*. The *Psychotria* genus comprises approximately 2,000 species native to humid lowland forests from northern Central America to central South America (Soares et al., 2017; Moraes et al., 2011). Many members of the Rubiaceae family are known to produce bioactive compounds (Moraes et al., 2011) but *P. viridis* deserves particular attention due to its capacity to produce the N,N-dimethyltryptamine (DMT). The DMT is a tryptamine with a potent effect as a psychoactive substance with entheogenic properties and feasible physiological and therapeutic roles in psychiatry (Barker, 2018; Orsolini et al., 2017). For instance, DMT has a particular interest in clinical trials as a medicine to treat depression, obsessive-compulsive disorders, the psychological impacts of terminal illnesses, prisoner’s recidivism, and substance abuse disorders (including alcohol and tobacco), among others (Barker, 2022; Cameron & Olson, 2018; Rodrigues, Almeida & Vieira-Coelho, 2019; Siegel et al., 2021).

For the Amazon rainforest’s indigenous peoples, *chacrona* is considered a “teacher plant” since its leaves are employed in the preparation of the ayahuasca tea, a sacred beverage used in ceremonial and spiritual rituals by the shamans and healers of the Amazon region. The ayahuasca brew is made of a decoction using the leaves of *chacrona* together with macerated pieces of the liana *Banisteriopsis caapi* (Spruce ex Griseb.) C.V.Morton (Malpighiaceae). *B. caapi*, popularly known as *mariri*, *cipó-mariri*, *caapi*, or *yagé*, produces psychoactive alkaloids from the beta-carboline class which act as monoamine oxidase inhibitors, potentiating the effects of the DMT in the traditional beverage (Morales-García et al., 2017; Santos et al., 2020).

The ayahuasca brew is also widely used in ceremonial and healing sessions of ayahuasca by some institutionalized Brazilian religions, such as “The Beneficent Spiritist Center União do Vegetal” (UDV) and “Santo Daime”, among others. Therefore, beyond being part of the extremely rich Amazonian biodiversity and showing significant medical potential (yet to be established), both *chacrona* and *mariri* are sacred plants to many traditional cultures and religions.

However, very little genomic information is available for ayahuasca plants. For instance, only the chloroplast genome of *B. caapi* is currently available (Ramachandran et al., 2018), and no genomics resources are available for *P. viridis*. Therefore, our research group started an initiative to unravel the genome biology and structure of the sacred Amazonian plants used for the ayahuasca brew. Our main goal is to generate the platinum genomes of *P. viridis* and *B. caapi* using cytogenetics, long reads sequencing technology, and chromosome conformation capture associated with short-read sequencing. In this work, we describe the first insights into *P. viridis* genome structure and evolution, describing the complete mitochondrial (mtDNA) and plastidial (ptDNA) as the first complete organellar genomic sequences for this species.

## Material and Methods

### Plant sampling, DNA extraction, and sequencing

Samples of *P. viridis* were collected in the Nucleo Menino Galante located at Serra da Cantareira (São Paulo) (23.380562 S, 46.58857 W) with the collaboration of União do Vegetal (approval number #23092021) and deposited at the Herbarium JABU from the Universidade Estadual Paulista (Unesp), campus Jaboticabal (voucher #3390). For the *P. viridis* genome sequencing, fresh leaves were collected after 24 h of dark treatment and submitted to flash freezing in liquid nitrogen, and stored at −80 °C in a super freezer. The plant samples were shipped to the Arizona Genomics Institute (Tucson, AZ, USA) where a high molecular weight (HMW) DNA extraction was performed using a modified CTAB protocol. The HMW DNA quality was checked for concentration on a Qubit dsDNA high-sensitivity assay, and absorbance ratios (260/280 and 260/230 nm) on a NanoDrop NP-1000 spectrophotometer (NanoDrop Technologies, Wilmington, DE, USA). The DNA size was validated by a Femto Pulse and pulse-field gel electrophoresis, and the purity was validated using restriction enzyme digestion test. The HMW DNA was sheared to the appropriate size range (10–30 kb) using a Covaris g-TUBE, followed by bead purification with PB Beads (PacBio, Menlo Park, CA, USA) and libraries construction according to the manufacturer’s protocol using a SMRTbell Express Template Prep Kit 2.0. Sequencing was performed on the PacBio Sequel IIe (CCS mode – HiFi reads) platform at Arizona Genomics Institute (Tucson, AZ, USA).

### Organelle genome assembly

We adopted a straightforward bioinformatics protocol to extract organellar-related reads from the sequenced reads. In the first step, we downloaded all ptDNA and mtDNA available sequences from GenBank Organelle Genome Resources (https://www.ncbi.nlm.nih.gov/genome/organelle/). For the ptDNA, the RefSeq fasta sequences were retrieved from the https://ftp.ncbi.nlm.nih.gov/refseq/release/plastid/ website, whereas the mtDNA RefSeq fasta sequences were obtained from the https://ftp.ncbi.nlm.nih.gov/refseq/release/mitochondrion/. In the second step, the fasta sequences of each RefSeq were concatenated and used to construct a custom Kraken2 (Wood, Lu & Langmead, 2019) database, according to the instructions provided at kraken2 GitHub and wiki (https://github.com/DerrickWood/kraken2/wiki/Manual#custom-databases). To facilitate the separation of the organellar HiFi reads from the sequencing reads, the ptDNA and mtDNA fasta sequences retrieved from the RefSeq had their “kraken:taxid” arbitrary set to “3702” for all ptDNA fasta sequences and “4530” for all mtDNA fasta sequences. In the third step, the Kraken v2.0 software was used to classify all sequenced reads using the custom database. Next, the “extract_kraken_reads.py” script from the KrakenTools (https://ccb.jhu.edu/software/krakentools/) was used to extract ptDNA and mtDNA related reads using the options:—include-children, —fastq-output, —taxid 3702 for ptDNA reads, and —taxid 4530 for mtDNA reads. Only organelle-related reads showing length >13 kb (N50 > 17 kb) and PHRED score >90 were used for the assembly. In the fourth step, each organelle was independently assembled with Flye v2.9 (Kolmogorov et al., 2019) using default parameters. In the final step, the “*get_organelle_from_assembly.py*” script from the GetOrganelle tool (Jin et al., 2020) was used to bait the ptDNA and mtDNA for each assembly graph generated (gfa file) using default parameters. The above-mentioned steps guaranteed a high-quality assembly showing average coverage of >500× for both organelles. The ptDNA and mtDNA candidate circular molecules were manually visualized and validated using the Bandage tool (Wick et al., 2015).

### Organellar genome annotation, and comparative analyses

The ptDNA and mtDNA were annotated using the GeSeq platform (Tillich et al., 2017). For ptDNA, the *Rubia cordifolia* (NC_047470.1) and *Coffea arabica* (NC_008535.1) were used as references. For the mtDNA, the Apocynaceae mitogenomes *Asclepias syriaca* (NC_022796), *Rhazya stricta* (NC_024293.1), *Cynanchum auriculatum* (NC_041494.1) and the Magnoliaceae *Liriodendron tulipifera* (NC_021152.1) were used as references. All sequences were obtained from the GenBank Organelle Genome Resources. For the evaluation of the potential transfer of Transposable Elements (TE) to the mitogenomes, the The Extensive *de novo* TE Annotator (EDTA) package was employed for *de novo* TE detection (Ou et al., 2019). Relics of TE-related genes were predicted with TEsorter using the genome mode (Zhang et al., 2022). The final annotation was manually verified and refined according to the known plant mtDNA genes previously reported (Mower, Sloan & Alverson, 2012), and the organellar genome maps were made with the combination of the OGDraw (Greiner, Lehwark & Bock, 2019) and DNAPlotter (Carver et al., 2009) tools. The OGDraw and DNAPlotter figures were combined and manually edited with Inkscape (https://inkscape.org).

ptDNA comparative analyses were carried out with IRscope (Amiryousefi, Hyvönen & Poczai, 2018). Phylogenomics analyses were estimated using the maximum likelihood (ML) approach with a concatenated matrix generated by the “catfasta2phyml.pl” script (https://github.com/nylander/catfasta2phyml) and composed of 70 ptDNA genes (*accD*, *atpA*, *atpB*, *atpE*, *atpF*, *atpH*, *atpI*, *ccsA*, *cemA*, *infA*, *matK*, *ndhA*, *ndhB*, *ndhC*, *ndhD*, *ndhE*, *ndhF*, *ndhG*, *ndhH*, *ndhI*, *ndhJ*, *ndhK*, *petA*, *petD*, *petG*, *petL*, *petN*, *psaA*, *psaB*, *psaC*, *psaI*, *psaJ*, *psbA*, *psbB*, *psbC*, *psbD*, *psbE*, *psbF*, *psbH*, *psbI*, *psbJ*, *psbK*, *psbL*, *psbM*, *psbN*, *psbT*, *psbZ*, *rbcL*, *rpl14*, *rpl16*, *rpl20*, *rpl22*, *rpl23*, *rpl32*, *rpl33*, *rpl36*, *rpoA*, *rpoB*, *rpoC1*, *rpoC2*, *rps2*, *rps3*, *rps4*, *rps7*, *rps8*, *rps11*, *rps14*, *rps15*, *rps16*, *rps18*) shared across 39 Rubiaceae species (MK203877, MK203879, MN883829, MT674513, MT674517, MT674520, MT674521, MT674522, MW548283, MZ425928, MZ958829, NC_008535, NC_028009, NC_028614, NC_030053, NC_034698, NC_036300, NC_036970, NC_041149, NC_044102, NC_047302, NC_047470, NC_049078, NC_049155, NC_049160, NC_050962, NC_053701, NC_053762, NC_053818, NC_054151, NC_057496, NC_057533, NC_057593, NC_057983, NC_057984, NC_057985, NC_058252, OK236359, OK236360). The ML tree was calculated using IQ-Tree 2 (Minh et al., 2020) with the best-of-fit model GTR+F+R3, according to AIC criteria (Akaike, 1974), with the software ModelFinder (Kalyaanamoorthy et al., 2017). The clade support was estimated with the ultrafast bootstrap (UFBoot) and SH-aLRT algorithms (Hoang et al., 2018) with 1,000 replicates. Tree rooting was based on the *Passiflora edulis* (NC_034285), *Vitis vinifera* (NC_007957) and *Arabidopsis thaliana* (NC_000932) species as outgroup. mtDNA comparative analyses were carried out with Sibelia (Minkin et al., 2013). The long reads have been aligned to the assembled mitogenomes with pbmm2 (https://github.com/PacificBiosciences/pbmm2), and the coverage was calculated with the script “wgscoverageplotter.jar” from the Java utilities for Bioinformatics (JVARKIT) (https://github.com/lindenb/jvarkit). All figures were manually edited with Inkscape (https://inkscape.org).

### Data availability

The *P. viridis* samples (GenBank BioSample SAMN26930853) were included in the Brazilian National System of Management of Genetic Heritage and Associated Traditional Knowledge (SisGen) under the access number #A3D39A2. The organellar genomes were deposited into GenBank, BioProject PRJNA819420, under the accession ON064099 for the ptDNA and ON064100 (mtDNA haplotype I) and ON968433 (mtDNA haplotype II) for the mtDNA.

## Results and discussion

### *Psychotria viridis* ptDNA structure and main features

**Figure 1:**
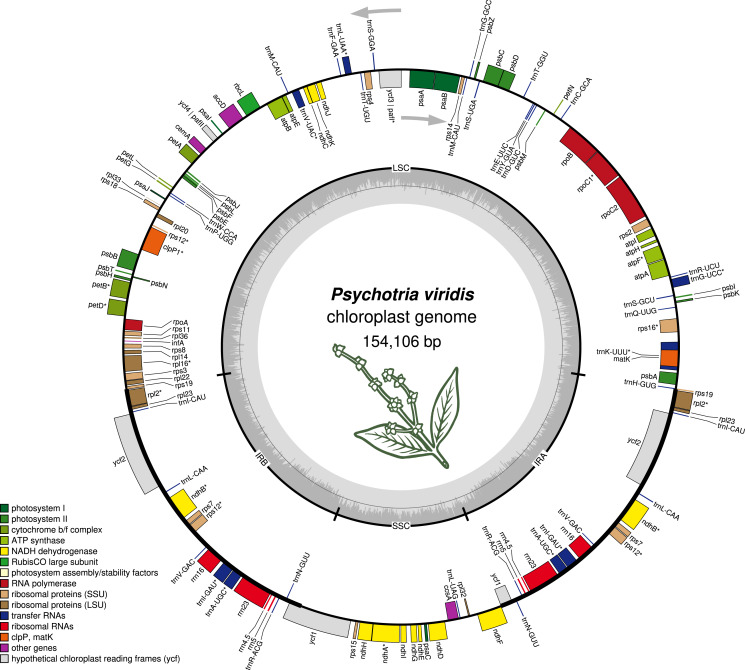
The structure of *Psychotria viridis* chloroplast genome. Genes shown on the outside of the map are transcribed clockwise, whereas genes on the inside are transcribed counter-clockwise. Genes are color-coded by their function in the legend.

**Figure 2:**
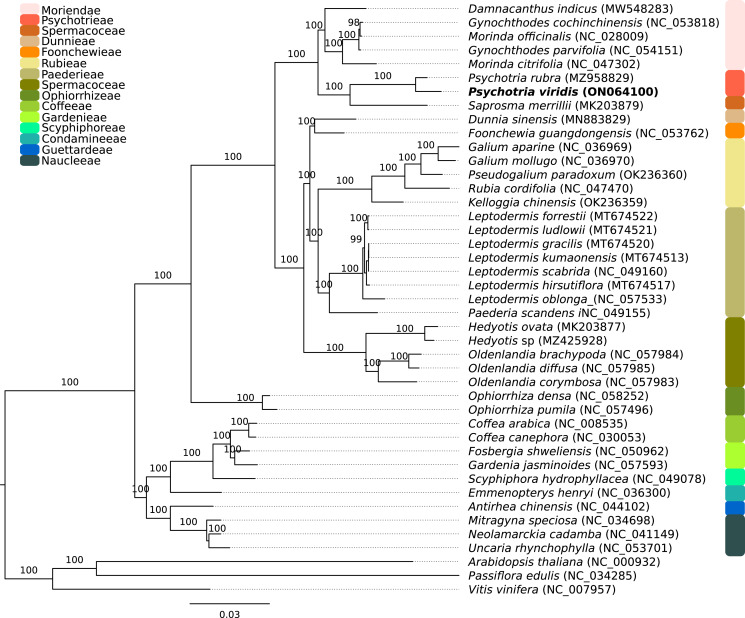
Phylogenomics tree of *Psychotria viridis* ptDNA and related plastids. Maximum likelihood tree of *Psychotria viridis* and related species within Rubiaceae using all shared ptDNA CDS. The *Arabidopsis thaliana* (NC_000932), *Passiflora edulis* (NC_034285), and *Vitis vinifera* (NC_007957) ptDNA were used as the outgroup.

The *P. viridis* ptDNA genome span 154,106 bp, exhibiting a GC content of 37.55% and a typical quadripartite structure encoding all known ptDNA genetic repertoire found in photosynthetic organisms (Fig. 1 and Table 1). Phylogenomic analyses based on ptDNA, clustered all tribes as monophyletic groups and placed *P. viridis* in the Psychotrieae tribe, sister to Spermacoceae and Moriendae tribes (Fig. 2). The close relationship among these tribes was already suggested based on nuclear and plastidial markers (Löfstrand, Razafimandimbison & Rydin, 2019; Razafimandimbison et al., 2020, 2017). The main differences are related to Spermacoceae clustered with Psychotrieae, while in the previous studies, the Moriendae is more closely related to Psychotrieae than Spermacoceae. Most of the Moriendae species shown in the Fig. 2 are known as medicinal plants in Southeast Asia, particularly in traditional Chinese medicine. However, among the three close-related tribes, only *P. viridis* is capable to produce alkaloids and DMT, making this species particular in terms of genetic content.

**Table 1:** List of genes encoded by the *Psychotria viridis* chloroplast genome.

Pathway	*Genes*
Photosystem I	*psaA, psaB, psaC, psaI, psaJ*
Photosystem II	*psbA, psbB, psbC, psbD, psbE, psbF, psbH, psbJ, psbK, psbL, psbM, psbN (same as psbf1), psbT, psbZ*
Cytochrome b/f complex	*petA, petB*^a^, *petD, petG, petL, petN*
ATP synthase	*atpA, atpB, atpE, atpF*^*a*^, *atpH, atpI*
NADH dehydrogenase	*ndhA*^a^, *ndhB (*×*2)*^a^, *ndhC, ndhD, ndhE, ndhF, ndhG, ndhH, ndhI, ndhJ, ndhK*
RubisCO large subunit	*rbcL*
RNA polymerase	*rpoA, rpoB, rpoC1*^a^, *rpoC2*
Ribosomal proteins (SSU)	*rps11, rps12, rps14, rps15, rps16*^a^, *rps18, rps19* (×2), *rps2, rps3, rps4, rps7* (×2), *rps8*
Ribosomal proteins (LSU)	*rpl14, rpl16*^a^, *rpl2 (*×*2)*^a^, *rpl20, rpl22, rpl23 (*×*2), rpl32, rpl33, rpl36*
Other genes	*infA*, *accD*, *ccsA*, *cemA*, *matK*, *clpP*^*b*^
hypothetical cp genes	*ycf1, ycf2 (*×*2), ycf3*^*b*^, *ycf4*
tRNAs	trnC-GCA, trnD-GUC, trnY-GUA, trnE-UUC, trnT-GGU, trnS-UGA, trnG-GCC, trnS-GGA, trnT-UGU, trnL-UAAa, trnF-GAA, trnV-GAC, trnM-CAU (×2), trnW-CCA, trnP-UGG, trnC-ACAa (×2), trnL-CAA (×2), trnI-GAU (×2)a, trnA-UGCa (×2), trnR-ACG (×2), trnN-GUU (×2), trnL-UAG, trnV-GAC (×2), trnH-GUG, trnK-UUUa, trnQ-UUG, trnS-GCU, trnG-UCCa, trnR-UCU.
rRNAs (duplicated copies)	*rrn23S* (×2), *rrn16S* (×2), *rrn5S* (×2), *rrn4.5S* (×2)

**Note:**

Genes are classified by pathways. Genes marked with the following signs are: (a) genes with a single intron; (b) genes with 2 introns; (×2) duplicated genes.

**Figure 3:**
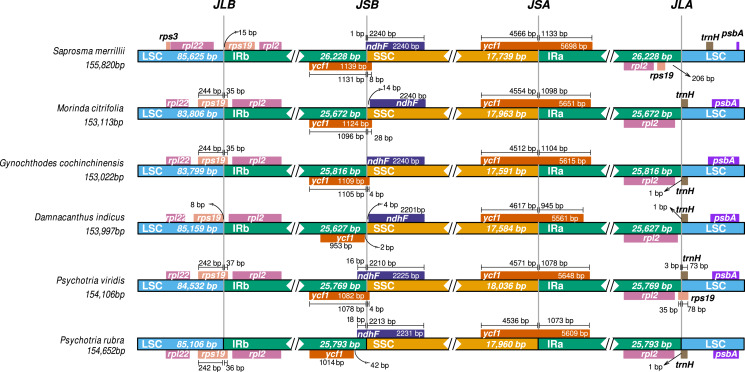
Comparative genomics of Rubiaceae plastids. Comparative genomics among different ptDNAs from the Rubiaceae family (Psychotrieae, Moriendae and Spermacoceae tribes), generated by the IRscope tool. JLB (IRb /LSC), JSB (IRb/SSC), JSA (SSC/IRa) and JLA (IRa/LSC) denote the junction sites between each corresponding region in the genome.

Comparative ptDNA analysis evidenced that Psychotrieae, Spermacoceae, and Moriendae tribes are exhibiting slight junction site variation between the plastid regions (IR-SSC-IR and IR-LSC-IR) (Fig. 3). For instance, a conserved junction site pattern is observed between the two available *Psychotria* ptDNA, where *P. rubra* and *P. viridis* exhibit the *ndhF* and *ycf1* genes delimiting the SSC borders (Fig. 3). The other junction site variation shown in Fig. 3 is common in other angiosperms and can be found in other plant clades (Amiryousefi, Hyvönen & Poczai, 2018; Chen et al., 2021; Li et al., 2021; Zhang et al., 2019).

### *Psychotria viridis* mtDNA presents a complex structure, composed of two main circular chromosomes that are arranged into several isoforms and sub-circles

The plant mitochondrial genomes exhibits a complicated mixture of physical configurations and sequences, thereby showing variable and dynamic connections, such as branched linear structures containing multigenomic concatemers together with rare circular forms (Kozik et al., 2019). Recently, it was proposed that analyses based on the assembly graphs approaches are valid for visualizing the actual complexity of the plant mtDNA’s variable forms within the mitochondria organelles (Fischer et al., 2022). Graph-based analyses can also provide sufficient evidence to avoid the naïve and erroneous conclusion that a single circular mtDNA molecule exists (Fischer et al., 2022; Kozik et al., 2019).

**Figure 4:**
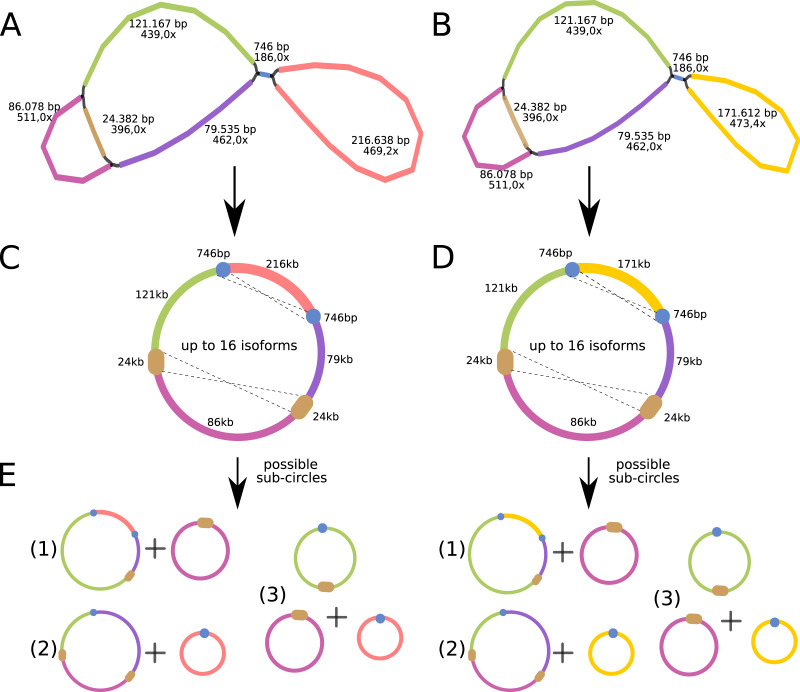
Mitochondrial genome analyses of *Psychotria viridis*. *Psychotria viridis* mitochondrial genome assembly graph shown by Bandage tool, disentangled assembly graph, and possible configurations (isoforms and sub-circles) of *P. viridis* mitogenome. (A) *P. viridis* mtDNA haplotype I is formed by six nodes and eight edges (N50 of 121-kb) showing median depth of 469× and estimated length of 527,800 bp. (B) *P. viridis* mtDNA haplotype II is formed by six nodes and eight edges (N50 of 121-kb) showing median depth of 473× and estimated length of 482,774 bp. (C) Disentangled assembly graph of *P. viridis* mtDNA haplotype I showing total size of 615,370 bp, the two major repeats (24-kb and 740 bp) and possible sites for homologous recombination events that may isomerize the circular molecules in up to 16 isoforms. (D) Disentangled assembly graph of *P. viridis* mtDNA haplotype II showing total size of 570,344 bp, the two major repeats (24-kb and 740 bp) and possible sites for homologous recombination events that may isomerize the circular molecules in up to 16 isoforms. (E) All possibles sub-circles that can be generated by repeat-mediated homologous recombination events.

The *P. viridis* mtDNA assembly graph presents two alternative and circular structures showing 44.37% of GC content, suggesting that heteroplasmy may be common in plants (Woloszynska, 2010). The mtDNA haplotype I spans 615,370 bp and contains one long (24 kb) repeat region, while the mtDNA haplotype II spans 570,344 bp and contains a smaller repeat region (740 bp) (Figs. 4A and 4B). Repeated regions can be the substrate for different homologous recombination or other recombination events that may symmetrically isomerize and rearrange the mitogenomes (Sloan, 2013). Therefore, these repeated regions may support the existence of a dynamic spectrum of up to 16 isoforms and up to eight different sub-circles arrangements for each mitogenome haplotype. All those variants probably coexist *in vivo* (Figs. 4C and 4D). Moreover, the 24 kb repeated region from the mtDNA haplotype I encodes for several mitochondrial genes, resulting in a redundant set of gene copies. This kind of complex structure is commonly found in other plant mitochondrial genomes and it is now considered more a rule than an exception that can result from repeat-mediated or other recombination events (Fischer et al., 2022; Kozik et al., 2019).

**Figure 5:**
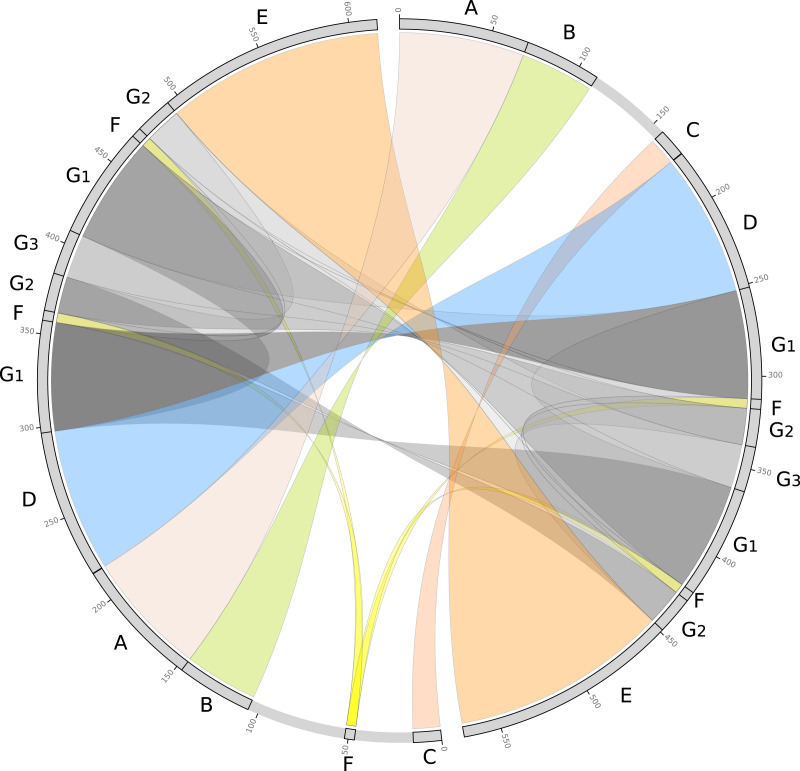
Comparative genomics analyses of the two mitochondrial haplotypes. Comparative alignment between *Psychotria viridis* mitochondrial genome haplotype I and II showing six large syntenic blocks (different colors) and the repeated regions (grey and yellow). The letters (A–G) denote the corresponding repeat regions.

We further evaluated the macrosynteny patterns, revealing that six large collinear blocks are shared among the mtDNA haplotype I and haplotype II (Figs. 5A–5E), in addition to the internal and shared repeats (Figs. 5F and 5G). The average nucleotide identity between both mitogenomes haplotypes is 100% between the shared collinear blocks. Moreover, alignment analyses indicated that 86% of haplotype I is covered by haplotype II, while 92% of haplotype I is covered by haplotype II. Therefore, there are 85,631 and 45,965 bp of exclusive regions for haplotype I and haplotype II, respectively.

### *Psychotria viridis* mtDNA gene content and main features

**Figure 6:**
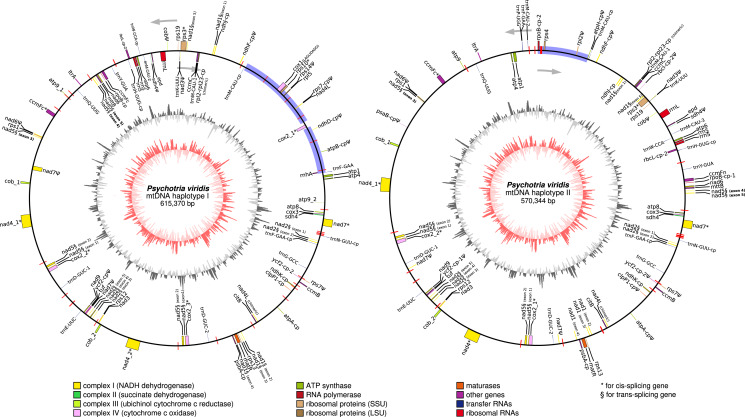
Circular representation of *Psychotria viridis* mitochondrial genome haplotype I and II. Genes shown on the outside of the map are transcribed clockwise, whereas genes on the inside are transcribed counter-clockwise. Genes are color-coded by their function in the legend. The 8-kb repeated region is highlighted in black boxes, exclusive regions are shown as blue retangles, and relics of TE-related genes are shown as purple lines. The inner circle represents the GC Skew (colors in red-scale), while the outside circle represents the GC% content (colors in gray-scale) (window size 1,000 bp, Step Size 100 bp).

In general, plant mtDNA genomes carry approximately 40 protein-coding genes, a variable set of tRNAs, and the insertion of ptDNA-related sequences as a product of lateral endosymbiotic gene transfer (Mower, Sloan & Alverson, 2012). The *P. viridis* mtDNA follows these trends, encoding most genes typically found in mitochondrial genomes (Fig. 6, Tables 2 and 3). The main finding is related to the absence of the *rps2*, *rps11*, *rpl2*, *rpl6*, *rpl10*, and *rpl16* genes, but the ribosomal genes are highly variable across the angiosperms (Mower, Sloan & Alverson, 2012). Additionally, the *sdh3* (Uniprot: P0DKI0 and GO:0000104), which encodes the succinate dehydrogenase cytochrome b subunit, is absent in both versions of the *P. viridis* mtDNAs, but also other plants mitogenomes (*i.e*., *Oryza sativa)*. On some plants like *Arabidopsis thaliana*, this gene has been integrated into the nuclear genome (Mower, Sloan & Alverson, 2012), supporting a potential function redundancy mechanism using nuclear homologous genes. Moreover, the *nad5* gene, which is formed by five exons, presents a duplicated copy of exons 1 and 2. Since *nad5* is processed by trans-splicing, this finding may suggest the existence of additional transcriptional paths responsible to join the spliceosome’s primary RNA transcripts into the *nad5* mRNA assembly.

**Table 2:** List of genes encoded by the *Psychotria viridis* mitochondrial genome haplotype I and haplotype II.

	Gene	*Haplotype I*	*Haplotype II*
Complex I (NADH dehydrogenase)	*nad1* §	+	+
	*nad2* §	+	+
	*nad3*	+ (+ Ψ partial)	+ (+ Ψ partial)
	*nad4* ‡	+ (×2)	+ (×2)
	*nad4L*	+	–
	*nad5* §	+ (×2)	+ (×2)
	*nad6*	+ (+ Ψ partial)	+ (+ Ψ partial)
	*nad7* ‡	+ (+2 Ψ partial)	+ (+2 Ψ partial)
	*nad9*	+	+
Complex II (succinate dehydrogenase)	*sdh3*	–	–
	*sdh4*	+ (+1 Ψ partial)	+ (+1 Ψ partial)
Complex III (ubiquinol cytochrome c reductase)	*cob*	+ (×2 + Ψ partial)	+ (×2 + Ψ partial)
Complex IV (cytochrome c oxidase)	*cox1* ‡	+ (LAGLIDADG)	–
	*cox2* ‡	+ (×3)	+ (×2)
	*cox3*	+	+
Complex V (ATP synthase)	*atp1*	+ (1,371 bp)	+ (1,776 bp)
	*atp4*	+	+
	*atp6*	+	+
	*atp8*	+	+
	*atp9*	+	+
Cytochrome c biogenesis	*ccmB*	+	+
	*ccmC*	+	+
	*ccmFc* ‡	+	+
	*ccmFn*	+	+
Ribosomal proteins (SSU)	*rps1*	+	+
	*rps2*	–	–
	*rps3* ‡	+	+
	*rsp4*	–	+
	*rps7*	Ψ	Ψ
	*rsp8*	–	–
	*rps10* ‡	+	–
	*rps11*	–	–
	*rps12*	+	+
	*rsp13*	+	+
	*rsp14*	Ψ	–
	*rps19*	+	+
Ribosomal proteins (LSU)	*rpl2*	–	Ψ
	*rpl5*	+	–
	*rpl6*	–	–
	*rpl10*	–	–
	*rpl16*	–	–
Matures	*matR*	+	+
Transport membrane proteins	*mttB*	+	+

**Note:**

Genes found in the two mitochondrial haplotypes in *P. viridis*. Genes are classified by pathways. Further information is provided as: Ψ for pseudogene; § for trans-splicing genes; and ‡ for intron-containing genes (cis-splicing).

**Table 3:** List of tRNA and rRNA genes encoded by the *Psychotria viridis* mitochondrial genome chromosome I and chromosome II.

	*Haplotype I*	*Haplotype II*
*rrnL*	+	+
*rrnS*	+	+
*rrn5*	+	+
*trnC* (GCA)	−	−
*trnC* (GCA)-cp	−	−
*trnD* (GUC)	+ (2×)	+ (2×)
*trnD* (GUC)-cp	−	−
*trnE* (UUC)	+	+
*trnF* (GAA)	+	+
*trnF* (GAA)-cp	+	+
*trnG* (GCC)	+	+
*trnH* (GUG)-cp	+	+
*trnI* (CAU)	−	−
*trnK* (UUU)	+	+
*trnM* (CAU)-cp	+	+
*trnM* (CAU)	+ (2×)	+ (3×)
*trnN* (GUU)-cp	+	−
*trnP* (UGG)	−	+
*trnP* (UGG)-cp	−	−
*trnQ* (UUG)	+	+
*trnR* (UCU)-cp	−	−
*trnS* (GCU)	−	−
*trnS* (GGA)-cp	−	−
*trnS* (UGA)	−	−
*trnW* (CCA)-cp	+	+
*trnY* (GUA)	+	+

**Note:**

Genes marked with 2× are duplicated genes and 3× are genes presenting three copies. Plus and minus represent the presence of the gene in the direct strand (+) or complementary-reverse strand (−).

Other remarkable features are related to the differences between the two mtDNA haplotypes, including: the presence of a LAGLIDADG homing endonuclease located between only the *cox1* intronic region of the mtDNA haplotype I, and an additional and chimeric version of *nad4L* (326 bp) identified in each of the mtDNA haplotypes. The LAGLIDADG homing endonuclease are cleaving enzymes that are generally encoded by introns, conferring intron mobility, and generally found in fungal mitochondrial genomes (Megarioti & Kouvelis, 2020). The presence of the LAGLIDADG in only mtDNA haplotype I suggests horizontal transfer from a fungal donor species, but still maintaining the *cox1* loci intact in the mtDNA haplotype II. Both chimeric *nad4L* share only the first 16 amino acids, including the start codon, located on the N-terminus of the protein. Interestingly, a closer alignment inspection on the *nad4L* chimeric using the UniProt Blast (Pundir et al., 2015), reveals that, while the N terminus of the protein (including the 16 amino acids above mentioned) matched to different plant mitochondrial genome (*i.e*., *Capsicum chinense*, *Nicotiana tabacum*, *Zea mays*, *Vitis vinifera*, *Glycine max*, and many others), the rest of the protein exhibits matches to ortholog proteins from Fungi, Bacteria and Nematoda (UniProt IDs: A0A2G9TNM8, A0A1Y1ZXS8, R6LZY0, A0A238BKX1, and A0A1Y2DMI6); thereby, supporting a chimeric structure possibly acquired by lateral transference.

Additionally, the *atp1* (1,776 bp) from haplotype II is 405 bp longer than its respective homolog located on haplotype I, indicating considerable differences at the protein C-terminal. Using the *Arabidopsis thaliana atp1* (Uniprot: P92549 – 1,521 bp) as a reference, we observed 95–96% of similarity across almost the entire proteins (from the start codon to the position 1,350), supporting that *P. viridis* exhibits indeed two different C-terminal domains of Atp1 protein. Therefore, it is possible to speculate that these differences may impact the electron transport complexes of the respiratory chain and ATP production in a unique way for this species. This hypothesis will need further investigation.

Slight differences in the *nad1* gene were also observed. For instance, the *nad1* from haplotype II lacks three nucleotides (AAT); however, it does not impact the protein translation, making both genes 99% identical, showing only two amino acids difference.

Several ptDNA sequences representing complete and fragmented genes, and pseudogenes were found dispersed across the mitogenomes of *P. viridis*, accounting for up to 7, 9 kb in each mtDNA haplotype (Table 4). Noteworthy, we have found a chimeric gene formed by fusion of the chloroplast *rpl2* and *rpl23* genes. Furthermore, the group II intron splicing reverse transcriptase gene (*ltrA*), which is commonly observed in the ptDNA of some Angiosperms, was found as a fragment in *P. viridis* mtDNA (Table 4), and no mitovirus-derived sequences were identified in *P. viridis* mtDNA. Interestingly we have found a gene encoding a potential plant aconitate/2-methylaconitate hydratase, which is commonly involved with the tricarboxylic acid cycle, the second stage of cellular respiration, and the glyoxylate cycle. In addition, a plant glyceraldehyde-3-phosphate dehydrogenase involved in the glycolysis, and an elongation factor related to the protein synthesis were identified in both mitogenomes haplotypes, thus, suggesting acquisition from the nuclear genome.

**Table 4:** Additional features encoded by the *Psychotria viridis* mitochondrial genome haplotype I and haplotype II.

mtDNA haplotype I	**-Partial ptDNA genes:** *ndhK*, *psbA*, *rbcL*, *rpoB*.
	**-Pseudogenes (ptDNA):** *atpA*, *atpB*, *atpF*, *clpP1*, *ndhD*, *ndhJ*, *rps7*, *ycf2*.
	**-Chimeric (ptDNA):** *rpl2-rpl23*.
	**-Other Genes:** Aconitate/2-methylaconitate hydratase, glyceraldehyde-3-phosphate dehydrogenase, Elongation factor, Group II intron-encoded protein LtrA, polyprotein, Ribonuclease H.
mtDNA haplotype II	**-Partial ptDNA genes:** *psaB, ndhJ, ndhK, rbcL*.
	**-Pseudogenes (ptDNA):** *atpA, atpF, clpP1, ndhF, psbA, rpoB, ycf2*.
	**-Chimeric (ptDNA):** *rpl2-rpl23*.
	**-Other Genes:** Aconitate/2-methylaconitate hydratase, glyceraldehyde-3-phosphate dehydrogenase, Elongation factor, Group II intron-encoded protein LtrA, polyprotein.

**Note:**

Tabulated differences on genes present in the two mitochondrial haplotypes.

Finally, genes encoding a ribonuclease H and an unknown polyprotein were identified in both mtDNA haplotypes. The presence of these genes is a clear indicator of a previous event of TEs migration from the nuclear to the mitochondrial genome. Indeed, several TEs scars were identified in *P. viridis* mtDNA, as revealed in Fig. 6, thus evidencing that these mobile elements helped to shape the structure of *chacrona*’s mitogenomes.

## Conclusions

The results presented here elicit primary insights into the genome biology and structure of the sacred and entheogenic plant *Psychotria viridis*, a main component of the ayahuasca beverage. While the ptDNA has shown a conserved gene content and organization when compared to other Rubiaceae, the *P. viridis* mitochondrial genome is heteroplasmic and has proven to be more complex, showing a dynamic mixture of forms. The complete versions of the ptDNA and mtDNA presented here are the first genomics resources for this species and can be further explored for other populational, comparative and evolutionary studies aiming to provide strategies to manage and conserve the biodiversity of the *chacrona*. Detailed analyses of the nuclear genome of *Psychotria viridis* are ongoing, and a complete description of its nuclear genome will be featured in future publications.

## Supplemental Information

10.7717/peerj.14114/supp-1 Supplemental Information 1 The plastid of *P. viridis* (ON064099), the mitochondrial haplotype I (ON064100), and haplotype II (ON968433).

10.7717/peerj.14114/supp-2 Supplemental Information 2 Mapping of the long-reads showing the average coverage of the *Psychotria viridis* mtDNA (haplotype I and II). **A.** Haplotype I showing mapped Reads: 109,577; alignments: 180,551; mapped bases: 428,859,712 bp; mean gap-compressed sequence identity: 95.97%; max mapped read length: 32,131 bp; mean mapped read length: 2,375 bp. **B.** Haplotype II showing mapped Reads: 107,257; alignments: 182,603; mapped bases: 405,925,449 bp; mean gap-compressed sequence identity: 96.01%; max mapped read length: 33,068 bp; mean mapped read length: 2,222 bp.

## Additional Information and Declarations

### Field Study Permissions

The following information was supplied relating to field study approvals (*i.e*., approving body and any reference numbers): Field experiments were approved by the Research Council of Centro Espírita Beneficente União do Vegetal (#23092021).

### DNA Deposition

The following information was supplied regarding the deposition of DNA sequences: The plastid of *P. viridis* (ON064099), the mitochondrial haplotype I (ON064100), and haplotype II (ON968433) are available at GenBank.

### Data Availability

The following information was supplied regarding data availability: The data is available at NCBI SRA: SRR20204395, SRP386416, PRJNA819420; ON064099, ON064100, and ON968433.

## References

[ref-1] Akaike H (1974). A new look at the statistical model identification. IEEE Transactions on Automatic Control.

[ref-2] Amiryousefi A, Hyvönen J, Poczai P (2018). IRscope: an online program to visualize the junction sites of chloroplast genomes. Bioinformatics.

[ref-3] Barker SA (2022). Administration of N,N-dimethyltryptamine (DMT) in psychedelic therapeutics and research and the study of endogenous DMT. Psychopharmacology.

[ref-4] Barker SA (2018). N, N-dimethyltryptamine (DMT), an endogenous hallucinogen: past, present, and future research to determine its role and function. Frontiers in Neuroscience.

[ref-5] Cameron LP, Olson DE (2018). Dark classics in chemical neuroscience: N, N-dimethyltryptamine (DMT). ACS Chemical Neuroscience.

[ref-6] Carver T, Thomson N, Bleasby A, Berriman M, Parkhill J (2009). DNAPlotter: circular and linear interactive genome visualization. Bioinformatics.

[ref-7] Chen N, Sha LN, Wang YL, Yin LJ, Zhang Y, Wang Y, Wu DD, Kang HY, Zhang HQ, Zhou YH, Sun GL, Fan X (2021). Variation in plastome sizes accompanied by evolutionary history in monogenomic triticeae (Poaceae: Triticeae). Frontiers in Plant Science.

[ref-8] Fischer A, Dotzek J, Walther D, Greiner S (2022). Graph-based models of the *Oenothera* mitochondrial genome capture the enormous complexity of higher plant mitochondrial DNA organization. NAR Genomics and Bioinformatics.

[ref-9] Greiner S, Lehwark P, Bock R (2019). OrganellarGenomeDRAW (OGDRAW) version 1.3.1: expanded toolkit for the graphical visualization of organellar genomes. Nucleic Acids Research.

[ref-10] Hoang DT, Vinh LS, Flouri T, Stamatakis A, von Haeseler A, Minh BQ (2018). MPBoot: fast phylogenetic maximum parsimony tree inference and bootstrap approximation. BMC Evolutionary Biology.

[ref-11] Jin JJ, Yu WB, Yang JB, Song Y, dePamphilis CW, Yi T-S, Li D-Z (2020). GetOrganelle: a fast and versatile toolkit for accurate de novo assembly of organelle genomes. Genome Biology.

[ref-12] Kalyaanamoorthy S, Minh BQ, Wong TKF, von Haeseler A, Jermiin LS (2017). ModelFinder: fast model selection for accurate phylogenetic estimates. Nature Methods.

[ref-13] Kolmogorov M, Yuan J, Lin Y, Pevzner PA (2019). Assembly of long, error-prone reads using repeat graphs. Nature Biotechnology.

[ref-14] Kozik A, Rowan BA, Lavelle D, Berke L, Schranz ME, Michelmore RW, Christensen AC (2019). The alternative reality of plant mitochondrial DNA: one ring does not rule them all. PLOS Genetics.

[ref-15] Li C, Cai C, Tao Y, Sun Z, Jiang M, Chen L, Li J (2021). Variation and evolution of the whole chloroplast genomes of *Fragaria* spp. (Rosaceae). Frontiers in Plant Science.

[ref-16] Löfstrand SD, Razafimandimbison SG, Rydin C (2019). Phylogeny of coussareeae (Rubioideae, Rubiaceae). Plant Systematics and Evolution.

[ref-17] Megarioti AH, Kouvelis VN (2020). The coevolution of fungal mitochondrial introns and their homing endonucleases (GIY-YIG and LAGLIDADG). Genome Biology and Evolution.

[ref-18] Minh BQ, Schmidt HA, Chernomor O, Schrempf D, Woodhams MD, von Haeseler A, Lanfear R (2020). IQ-TREE 2: new models and efficient methods for phylogenetic inference in the genomic era. Molecular Biology and Evolution.

[ref-19] Minkin I, Patel A, Kolmogorov M, Vyahhi N, Pham S, Darling A, Stoye J (2013). Sibelia: a scalable and comprehensive synteny block generation tool for closely related microbial genomes. Algorithms in Bioinformatics, Lecture Notes in Computer Science.

[ref-40] Moraes TMS, Rabelo GR, Alexandrino CR, Silva Neto SJ, Cunha M (2011). Comparative leaf anatomy and micromorphology of Psychotria species (Rubiaceae) from the Atlantic Rain forest. Acta Botanica Brasilica.

[ref-20] Morales-García JA, de la Fuente Revenga M, Alonso-Gil S, Rodríguez-Franco MI, Feilding A, Perez-Castillo A, Riba J (2017). The alkaloids of *Banisteriopsis caapi*, the plant source of the Amazonian hallucinogen Ayahuasca, stimulate adult neurogenesis in vitro. Scientific Reports.

[ref-21] Mower JP, Sloan DB, Alverson AJ, Wendel JF, Greilhuber J, Dolezel J, Leitch IJ (2012). Plant mitochondrial genome diversity: the genomics revolution. Plant Genome Diversity.

[ref-22] Orsolini L, St John-Smith P, McQueen D, Papanti D, Corkery J, Schifano F (2017). Evolutionary considerations on the emerging subculture of the E-psychonauts and the novel psychoactive substances: a comeback to the shamanism?. Current Neuropharmacology.

[ref-23] Ou S, Su W, Liao Y, Chougule K, Agda JRA, Hellinga AJ, Lugo CSB, Elliott TA, Ware D, Peterson T, Jiang N, Hirsch CN, Hufford MB (2019). Benchmarking transposable element annotation methods for creation of a streamlined, comprehensive pipeline. Genome Biology.

[ref-25] Pundir S, Magrane M, Martin MJ, O’Donovan C (2015). Searching and navigating UniProt databases. Current Protocols in Bioinformatics.

[ref-26] Ramachandran P, Zhang N, McLaughlin WB, Luo Y, Handy S, Duke JA, Vasquez R, Ottesen A (2018). Sequencing the vine of the soul: full chloroplast genome sequence of *Banisteriopsis caapi*. Genome Announcements.

[ref-27] Razafimandimbison SG, Kainulainen K, Senterre B, Morel C, Rydin C (2020). Phylogenetic affinity of an enigmatic Rubiaceae from the Seychelles revealing a recent biogeographic link with Central Africa: gen. nov. Seychellea and trib. nov. Seychelleeae. Molecular Phylogenetics and Evolution.

[ref-28] Razafimandimbison SG, Kainulainen K, Wikström N, Bremer B (2017). Historical biogeography and phylogeny of the pantropical Psychotrieae alliance (Rubiaceae), with particular emphasis on the Western Indian Ocean Region. American Journal of Botany.

[ref-29] Rodrigues AV, Almeida FJ, Vieira-Coelho MA (2019). Dimethyltryptamine: endogenous role and therapeutic potential. Journal of Psychoactive Drugs.

[ref-30] Santos BWL, de Oliveira RC, Sonsin-Oliveira J, Fagg CW, Barbosa JBF, Caldas ED (2020). Biodiversity of β-carboline profile of *Banisteriopsis caapi* and ayahuasca, a plant and a brew with neuropharmacological potential. Plants.

[ref-31] Siegel AN, Meshkat S, Benitah K, Lipsitz O, Gill H, Lui LMW, Teopiz KM, McIntyre RS, Rosenblat JD (2021). Registered clinical studies investigating psychedelic drugs for psychiatric disorders. Journal of Psychiatric Research.

[ref-32] Sloan DB (2013). One ring to rule them all? Genome sequencing provides new insights into the ‘master circle’ model of plant mitochondrial DNA structure. New Phytologist.

[ref-39] Soares DB, Duarte LP, Cavalcanti AD, Silva FC, Braga AD, Lopes MT, Takahashi JA, Vieira-Filho SA (2017). Psychotria viridis: Chemical constituents from leaves and biological properties. Anais da Academia Brasileira de Ciencias.

[ref-33] Tillich M, Lehwark P, Pellizzer T, Ulbricht-Jones ES, Fischer A, Bock R, Greiner S (2017). GeSeq – versatile and accurate annotation of organelle genomes. Nucleic Acids Research.

[ref-34] Wick RR, Schultz MB, Zobel J, Holt KE (2015). Bandage: interactive visualization of de novo genome assemblies. Bioinformatics.

[ref-35] Woloszynska M (2010). Heteroplasmy and stoichiometric complexity of plant mitochondrial genomes—though this be madness, yet there’s method in’t. Journal of Experimental Botany.

[ref-36] Wood DE, Lu J, Langmead B (2019). Improved metagenomic analysis with Kraken 2. Genome Biology.

[ref-37] Zhang R, Ge F, Li H, Chen Y, Zhao Y, Gao Y, Liu Z, Yang L (2019). PCIR: a database of plant chloroplast inverted repeats. Database.

[ref-38] Zhang R-G, Li G-Y, Wang X-L, Dainat J, Wang Z-X, Ou S, Ma Y (2022). TEsorter: an accurate and fast method to classify LTR-retrotransposons in plant genomes. Horticulture Research.

