# Supplementary figures and images for: The complete organellar genomes of the entheogenic plant Psychotria viridis (Rubiaceae), a main component of the ayahuasca brew
Source: PeerJ. 2022 Oct 18;10:e14114. doi: 10.7717/peerj.14114 (PMC9586082; doi:10.7717/peerj.14114)

A

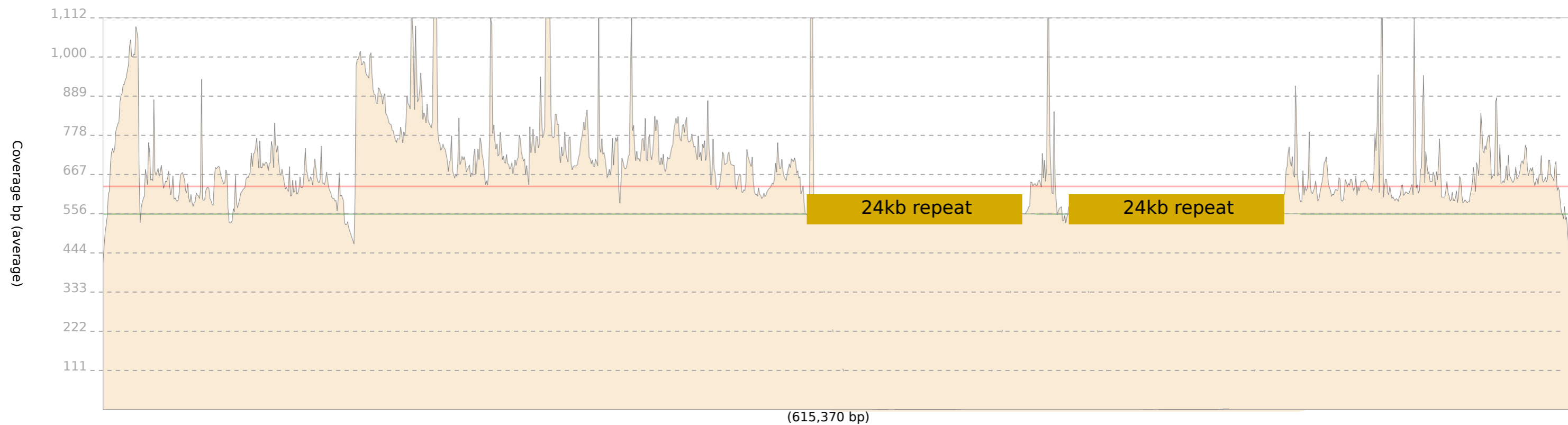

B

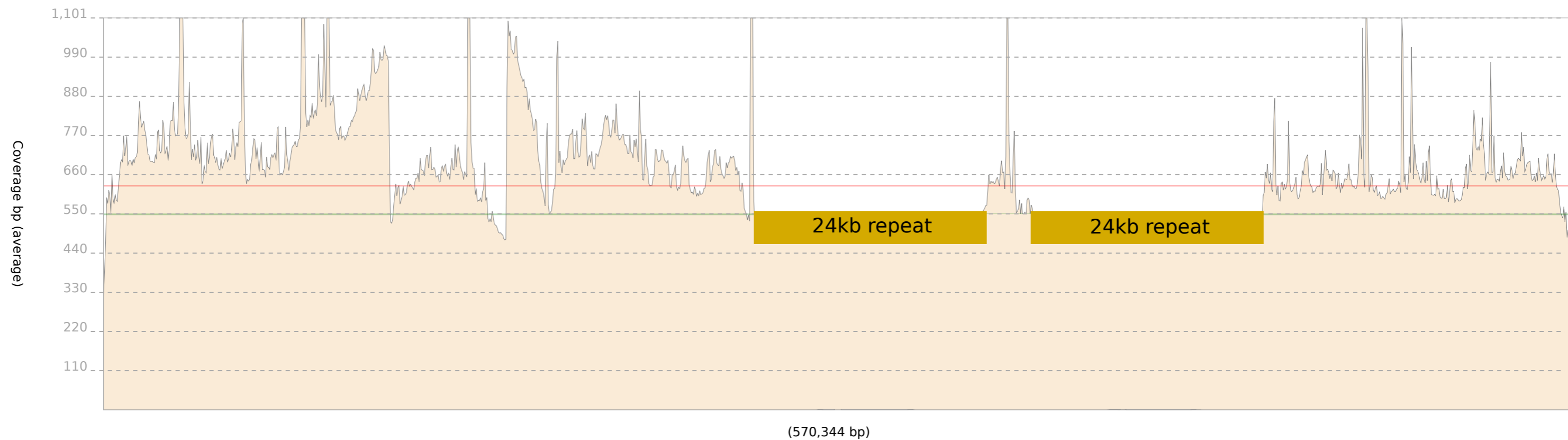

Supplement: Supplemental Information 2 — A. Haplotype I showing mapped Reads: 109,577; alignments: 180,551; mapped bases: 428,859,712 bp; mean gap-compressed sequence identity: 95.97%; max mapped read length: 32,131 bp; mean mapped read length: 2,375 bp. B. Haplotype II showing mapped Reads: 107,257; alignments: 182,603; mapped bases: 405,925,449 bp; mean gap-compressed sequence identity: 96.01%; max mapped read length: 33,068 bp; mean mapped read length: 2,222 bp. [file peerj-10-14114-s002.pdf]
